# Divergent Mechanisms of Internode Elongation in Response to Far-Red in Two Rose Genotypes

**DOI:** 10.3390/plants14071115

**Published:** 2025-04-03

**Authors:** Laurent Crespel, Camille Le Bras, Bénédicte Dubuc, Maria-Dolores Perez-Garcia, Esther Carrera, Aurélia Rolland, Rémi Gardet, Soulaiman Sakr

**Affiliations:** 1 Institut Agro, University Angers, INRAE, IRHS, SFR QUASAV, 49000 Angers, France; camille.lebras@institut-agro.fr (C.L.B.); benedicte.dubuc@univ-angers.fr (B.D.); maria-dolores.perez-garcia@institut-agro.fr (M.-D.P.-G.); remi.gardet@institut-agro.fr (R.G.); soulaiman.sakr@institut-agro.fr (S.S.); 2 Instituto de Biologia Molecular y Celular de Plantas (IBMCP), CSIC-Universidad Politécnica de Valencia, 46022 Valencia, Spain; ecarrera@ibmcp.upv.es; 3 University Angers, SFR QUASAV, 49000 Angers, France; aurelia.rolland@univ-angers.fr

**Keywords:** far-red, cell division, cell elongation, gibberellin, jasmonic acid, phenotypic stability, rose

## Abstract

The quality of potted ornamental plants depends on their architecture, which should be compact and branched. Among the techniques for controlling this architecture, LED lighting, by manipulating light quality, offers an effective means of regulating elongation and branching. In rose, the addition of far-red (FR) light stimulated branching but induced excessive stem elongation, i.e., internode elongation. However, some varieties remained insensitive to this effect, demonstrating phenotypic stability. This study investigated the underlying mechanisms of internode elongation in response to FR in two rose cultivars, ‘The Fairy’ (TF) and Knock Out^®^ Radrazz (KO), selected for their respective architectural plasticity and stability to FR. In TF, exposure to FR induced elongation of internodes, driven by cell division, with an increase in gibberellin A4 (GA4) level and a reduction in defense hormones (salicylic acid and jasmonic acid; JA). In contrast, in KO, exposure to FR did not induce internode elongation but caused cell elongation. This effect was accompanied by a reduction in cell number, modulated by hormonal changes (particularly GA4 and JA) and the inhibition of *Block of cell proliferation 1*, thereby limiting cell division. A deeper understanding of the mechanisms underlying architectural stability might lead to developing strategies to produce compact, branched plants, regardless of environmental conditions.

## 1. Introduction

The quality of potted ornamental plants is primarily determined by their shape, which must be both compact and well branched [1,2]. This shape results from the spatial arrangement of various organs according to species-specific rules. This complex architecture, particularly in ornamental shrubs such as rose and camellia, depends on genetic and environmental factors [3,4,5]. While architectural characteristics, such as the number of branches, are moderate to highly heritable, others, such as stem length and inclination, are more influenced by the environment [6,7,8,9,10,11].

Ornamental potted plants are typically grown in a greenhouse during the winter to be sold in the spring. These growing conditions, characterized by a high planting density, promote plant etiolation, which refers to excessive stem elongation accompanied by reduced branching, thus deviating from the desired quality standard: obtaining a compact and branched plant. Etiolation results from the plant’s shade-avoidance response, triggered via a modification in the light spectrum, i.e., a decrease in the red (R; 600–700 nm)/far-red (FR; 700–800 nm) ratio and the amount of blue light (B; 400–500 nm), perceived by the plant through various photoreceptors [12,13].

To promote branching and limit stem elongation, horticulturists have traditionally employed techniques such as “pinching” and chemical growth regulators. However, due to their economic cost and harmful environmental impacts, alternatives based on environmental control have been developed, including water restriction [7,14] and mechanical stimulation [15,16]. More recently, with the advancement of light-emitting diode (LED) technology, another approach focusing on manipulating light quality has also been explored [17]. In greenhouse-grown poinsettia, the use of LED lighting-emitting R and B was shown to increase the R/FR ratio and the amount of B, thereby reducing stem elongation [18]. In rose, this effect was enhanced by an increased proportion of B [19]. The decrease in the R/FR ratio, through the addition of FR, on the other hand, stimulated branching in chrysanthemum and rose grown in a controlled environment [20,21,22]. However, in rose, this addition of FR led to plant etiolation, thus affecting their quality. This excessive stem elongation might result from an improvement in the plant’s photosynthetic capabilities, increasing its carbon status, which is essential for growth [22]. In other species, such as Arabidopsis, sunflower, and lisianthus, this has been attributed to hormonal changes, particularly an increase in gibberellin (GA) concentrations, especially the biologically active forms, gibberellin A1 (GA1) and A4 (GA4), as well as auxins, which are hormones known to stimulate cell division and elongation [23,24,25,26,27,28,29]. Jasmonic acid (JA), another hormone known to activate the plant’s defense mechanisms, is also thought to be involved. Indeed, in Arabidopsis mutants deficient in JA synthesis and signaling, excessive hypocotyl elongation was observed under a low R/FR ratio [30]. Moreover, GAs and JA act antagonistically to regulate growth. Under FR exposure, GAs inhibit JA signaling [31]. As a result, the plant might reallocate its carbon resources, initially intended for defense, toward elongation.

However, in rose, as in chrysanthemum, a variety-specific response to light quality was observed, with certain varieties showing no response to the addition of FR [21,22,32]. This lack of response, i.e., this architectural stability, was correlated with an increase in the abscisic acid (ABA) concentration in the leaves, which might inhibit the stimulating effect of FR on photosynthesis and, consequently, on the carbon status required for stem elongation [22]. A better understanding of the physiological and molecular mechanisms underlying this architectural stability might pave the way for a new approach to obtaining plants with desired branching patterns and compactness, regardless of environmental conditions.

Therefore, this study aimed to explore the mechanisms underlying this stability in stem elongation, i.e., internode length, a crucial process for controlling compactness, in rose using a multidisciplinary approach: histological, biochemical (hormone concentrations), and molecular (transcriptomic analysis by RNA-seq), focusing on two varieties, ‘The Fairy’ (TF) and Knock Out^®^ Radrazz (KO), chosen respectively for their architectural plasticity and stability to FR.

## 2. Results

### 2.1. Effects of Far-Red (FR) on Internode Elongation in ‘The Fairy’ (TF) and Knock Out^®^ Radrazz (KO)

**Figure 1:**
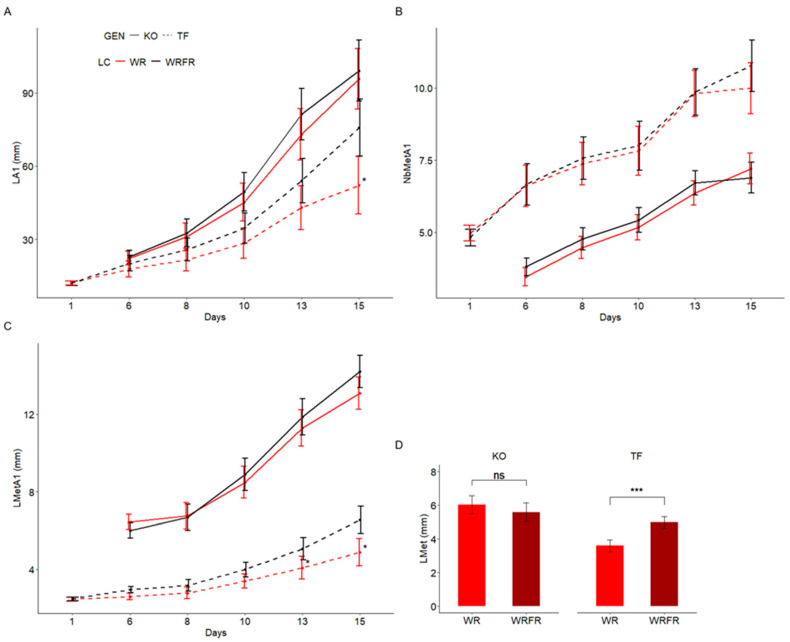
Kinetics of construction of the order 1 axis for Knock Out^®^ Radrazz (KO) and ‘The Fairy’ (TF) grown in a climate chamber under two light conditions: WR and WRFR. Kinetics of the elongation of the order 1 axis (LA1, length of the order 1 axis (**A**)). Kinetics of metamer emission by the order 1 axis (NbMetA1, number of metamers of the order 1 axis (**B**)). Kinetics of the elongation of metamers of the order 1 axis (LMetA1, length of metamers of the order 1 axis (**C**)). Least-square means (LS means) of the length of the metamer underlying the apex of the order 1 axis (LMet) on the 13th day of cultivation (**D**). Error bars represent SEMs (*n* = 30–36). For each genotype (GEN), asterisks indicate statistically significant differences between the light conditions (LC; Tukey’s test, *** *p* < 0.0001; ** *p* < 0.001; * *p* < 0.05; ns, non-significant).

As expected, in the presence of FR, TF exhibited etiolation, while KO remained unaffected. This etiolation effect was noticeable from (1) the 15th day of cultivation for the length of the order 1 axis (LA1), with a 45.3% increase (Figure 1A); and (2) from the 13th day of cultivation for the length of the metamer of the order 1 axis (LMetA1), with a 24.0% increase (Figure 1C). However, no effect was observed on the number of metamers of the order 1 axis (NbMetA1; Figure 1B). For biochemical and transcriptomic analyses, the internode located just below the apex was collected on the 13th day of cultivation. Under the effect of FR, its length increased by 38.2% in TF, whereas no difference was observed in KO (Figure 1D).

**Figure 2:**
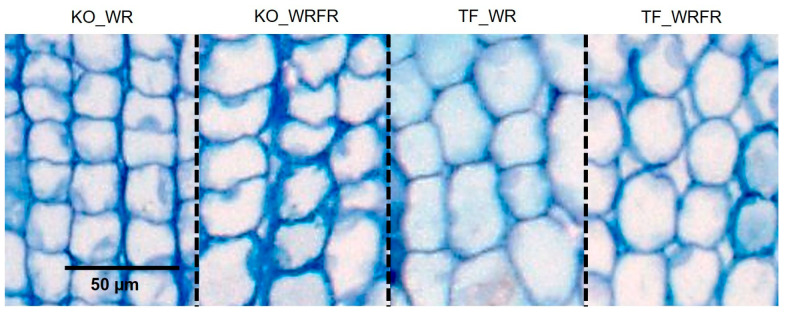
Longitudinal section of the fourth internode underlying the apex of the order 1 axis for Knock Out^®^ Radrazz (KO) and ‘The Fairy’ (TF) grown in a climate chamber for 14 days under two light conditions: WR and WRFR.

A histological analysis of longitudinal sections of the internodes was conducted to determine whether the internode elongation resulted from an increase in cell division, cell expansion, or a combination of both. In TF, no cell elongation was observed in response to FR (Table 1, Figure 2). Instead, internode elongation was solely driven by an increase in cell number (estimated at 34.7%; Table 1). In contrast, for KO, histological analysis revealed a 10.7% increase in cell length in response to FR (Table 1, Figure 2). However, this increase, coupled with a 16.6% decrease in cell number, resulted in the absence of internode elongation (Table 1).

### 2.2. Hormonal Changes Associated with FR-Induced Internode Elongation in TF and KO

**Figure 3:**
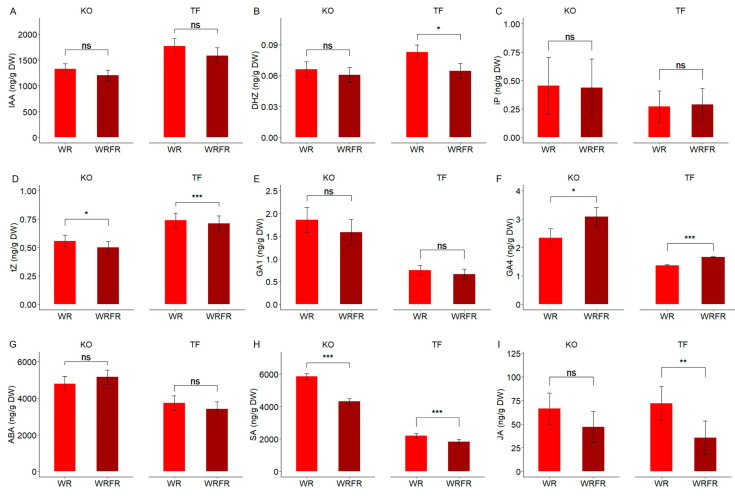
Least-square means (LS means) of hormone concentrations in the internode underlying the apex of the order 1 axis for Knock Out^®^ Radrazz (KO) and ‘The Fairy’ (TF) grown in a climate chamber for 14 days under two light conditions: WR and WRFR. Indole-3-acetic acid (IAA; (**A**)). Dihydrozeatin (DHZ; (**B**)). Isopenteniladenine (iP; (**C**)). t-zeatin (tZ; (**D**)). Gibberellin A1 (GA1; (**E**)). Gibberellin A4 (GA4; (**F**)). Abscisic acid (ABA; (**G**)). Salicylic acid (SA; (**H**)). Jasmonic acid (JA; (**I**)). Error bars represent SEMs (*n* = 3). For each genotype, asterisks indicate statistically significant differences between the light conditions (Tukey’s test, *** *p* < 0.0001; ** *p* < 0.001; * *p* < 0.05; ns, non-significant).

In TF, the elongation of internodes observed under the effect of FR was associated with an increase in gibberellin A4 concentration (GA4; +21.4%; Figure 3F) and a decrease in dihydrozeatin (DHZ) concentration (−21.8%; Figure 3B) in the internode. A slight reduction in the concentration of trans-zeatin (tZ) was also noted, although this was minimal (−3.4%; Figure 3D). Another group of hormones, salicylic acid (SA) and jasmonic acid (JA), was similarly affected. In response to FR, their concentration decreased by 16.4% and 50.6%, respectively (Figure 3H,I).

A similar trend was observed in KO, even though no internode elongation was noted under FR. The concentration of GA4 increased by 32.0% in the internode (Figure 3F), while concentrations of tZ and SA decreased by 10.4% and 26.2%, respectively (Figure 3D,H). A decrease in JA concentration was also observed (−28.9%, Figure 3I); however, this was not significant (Tukey test, *p* = 0.058).

For the other hormones, including indole-3-acetic acid (IAA), isopentenyladenine (iP), GA1, and abscisic acid (ABA), no effect of FR was observed on their concentration in the internode, regardless of the variety (Figure 3A,C,E,G).

### 2.3. Gene Expression Changes in the Internode in Response to FR in TF and KO

In TF, only the expression of four genes in the internode was affected by FR, with an adjusted *p*-value < 0.05 (Table 2). Among these genes, two are involved in the plant’s defense responses: the transcription factor *MYB73* and the serine/threonine kinase receptor *RBK2*. Their expression was inhibited in the presence of FR, with log_2_ fold changes (FC) ranging from −1.2 to −1.0 (Table 2).

Similarly, only three genes were differentially expressed in KO under FR, with an adjusted *p*-value < 0.05 (Table 2). However, increasing the significance threshold to *p* < 0.1 identified four additional genes (Table 2). Among these seven genes, three are involved in various cellular processes, ranging from cell-cycle regulation (*Block of Cell Proliferation 1*; *BOP1*) to cell expansion (*Walls Are Thin 1*; *WAT1*) and cell differentiation (*Poltergeist-like*; *PLL4*). Two others are involved in gibberellin catabolism: *Gibberellin 2-beta-dioxygenase 1* and *8* (*GA2ox1* and *GA2ox8*). Except for *RhWAT1*, the expression of these genes was inhibited under FR, with log_2_ FC ranging from −1.0 to −1.8 (Table 2). In contrast, the expression of *RhWAT1* was significantly increased (log_2_ FC = 2.5; Table 2).

**Figure 4:**
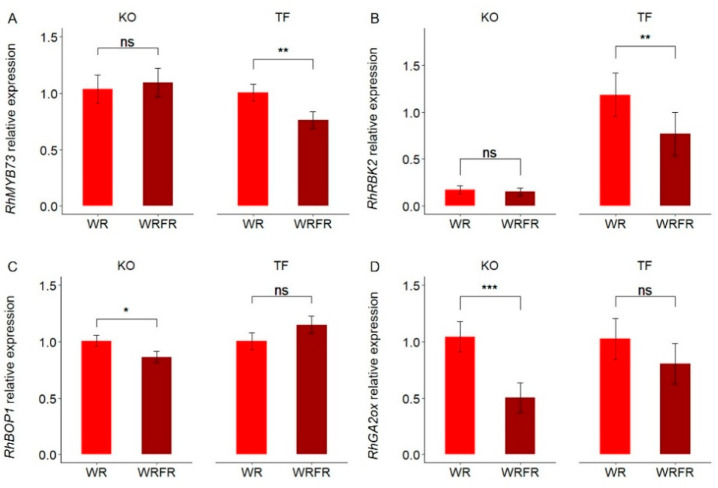
qRT-PCR validation of RNA-seq data. Least-square means (LS means) of relative expression of *RhMYB73* (**A**), *RhRBK2* (**B**), *RhBOP1* (**C**), and *RhGA2ox* (**D**) genes in the internode underlying the apex of the order 1 axis for Knock Out^®^ Radrazz (KO) and ‘The Fairy’ (TF) grown in a climate chamber for 13 days under two light conditions: WR and WRFR. Error bars represent SEMs (*n* = 3). For each genotype, asterisks indicate statistically significant differences between the light conditions (Tukey’s test, *** *p* < 0.0001; ** *p* < 0.001; * *p* < 0.05; ns, non-significant).

To ensure the validity and reliability of the transcriptomic analysis results, the expression levels of four differentially expressed genes (*RhMYB73*, *RhRBK2*, *RhBOP1,* and *RhGA2ox*) in both TF and KO were verified via qRT-PCR. The same expression profiles were observed, showing a decrease in gene expression under the effect of FR (Figure 4).

## 3. Discussion

‘The Fairy’ (TF) and Knock Out^®^ Radrazz (KO) responded differently to far-red (FR), highlighting specific regulatory mechanisms of internode elongation. For TF, the elongation of internodes might primarily result from a redirection of carbon resources initially allocated to defense mechanisms toward growth processes. In contrast, for KO, the inhibition of cell division and differentiation processes, coupled with the stimulation of cell elongation, might prevent or limit internode elongation.

In TF, as previously shown by [22], an elongation of the stem, specifically the internodes, was observed in response to FR, with an overall increase of 24.0%. Regarding the internode located just below the apex, this increase even reached 38.2%. This elongation might result, at least in part, from an increase in the number of cells, i.e., cell division, and may not involve cell elongation, contrary to what was initially reported in bean [23] and in lisianthus [28]. As observed in bean, sunflower, and Arabidopsis, elongation in TF was accompanied by an increase in gibberellin (GA) concentrations, especially the biologically active form, gibberellin A4 (GA4; 21.4%), which is a positive regulator of both cell division and elongation [23,26,27,33]. In parallel, a decrease in cytokinin (CK) concentrations, specifically the active form, dihydrozeatin (DHZ; −21.8%), was observed, as previously reported in sunflower [26]. In tomato, it was associated with a decrease in branching [34], as CKs are inducers of shoot branching [35]. However, [36] showed that an increase in DHZ concentration inhibited the elongation of pea stem segments cultured in vitro.

These hormonal modifications were also accompanied by a significant reduction in the concentrations of two defense hormones, salicylic acid (SA; −16.4%) and jasmonic acid (JA; −50.6%). This decrease, widely reported in the literature, leads to a decline in the plant’s defensive capacities [37,38]. In line with this, there was an inhibition of the expression of two genes, *RhMYB73* and *RhRBK2*, involved in the plant’s defense mechanisms. MYB73 is a transcription factor belonging to the R2-R3-MYB subfamily, known to enhance resistance to *Bipolaris oryzae* in Arabidopsis and to *Botryosphaeria dothidea* in apple by increasing SA concentration [39,40]. RBK2 is a cytosolic kinase belonging to the RLCK-VIb receptor family in Arabidopsis, whose expression is associated with the hypersensitive response triggered by *Phytophthora infestans* and *Botrytis cinerea* [41]. It is, therefore, more likely that the overall immunity of TF was reduced in response to FR. In addition to their role in plant defenses, JA is also known to inhibit growth, particularly root and hypocotyl elongation in Arabidopsis and internode elongation in rice, by hindering cell division [30,31,38,42,43,44,45]. It is thought to interact with GAs in a coordinated but antagonistic manner to regulate growth. Under conditions of a low R/FR ratio, GAs may inhibit the JA signaling by promoting the degradation of DELLA proteins (GA signaling repressors) and enhancing the stability of JASMONATE ZIM DOMAIN (JAZ) proteins (JA signaling repressors), as well as their interaction with MYC2 (a transcription factor that mediates JA signaling), thereby rendering it inactive [31,46,47]. It is, therefore, more likely that the plant redirected the allocation of carbon resources initially intended for defense toward elongation. This redistribution, coupled with an increased photosynthetic capacity [22], might promote growth and, as a result, the elongation of TF internodes in response to FR.

In KO, although internode elongation was not observed, FR caused notable histological changes, particularly a 10.7% increase in cell length, accompanied by a reduction in cell number (−16.6%). This cell extension correlated with an increase in GA concentrations, especially in GA4 (+32.0%), consistent with [23] and [28] on bean and lisianthus, respectively. However, this correlation was not observed in the case of TF. Recent studies suggest that GA signaling might play a crucial role in low R/FR or shade-induced stem elongation, as demonstrated in Chinese red pine [29] and proposed for sorghum [48]. KO is expected to be more sensitive to GAs than TF, as observed between dwarf and conventional-height durum wheat varieties [49]. Exogenous GA application treatments could help confirm this and provide a deeper understanding of the genotype-specific response to FR. Furthermore, [22] observed that the concentration of sucrose and soluble sugars in KO stems was higher than in TF, thereby providing the necessary energy and carbon for cell growth.

In KO, the elevated level of GA was accompanied by the inhibition of the genes encoding two GA catabolizing enzymes (*GA2ox1* and *GA2ox8*). Interestingly, the expression of *Walls Are Thin 1* (*WAT1*), which is involved in auxin signaling and the formation of secondary cell walls in wood fibers, was stimulated [50]. As a result, inhibiting *WAT1* limited cell elongation, leading to reduced stem length in Arabidopsis [50] and a dwarf phenotype characterized by short internodes in cotton [51]. While these factors could explain variations in cell length, others might also contribute to reducing cell number. Among the differentially expressed genes, two, *Block of Cell Proliferation 1* (*BOP1*) and *Poltergeist-like* (*PPL4*), involved in cell division and differentiation processes, were downregulated in response to FR. *BOP1* plays a crucial role in regulating these processes by controlling ribosome biogenesis, which is essential for cell proliferation [52,53,54,55]. Its impairment was associated with growth abnormalities in Arabidopsis, strawberry, and cotton, such as reduced root, hypocotyl elongation, and leaf deformities [53,54,55]. In Arabidopsis leaves, this inhibition limited cell expansion by limiting the cells’ ability to divide and elongate properly [53]. In both Arabidopsis and cotton, the inhibition of *BOP1* was also accompanied by an increase in the expression of genes related to JA biosynthesis, as well as an increase in its concentration [53,55]. Similarly, in KO, although a non-significant decrease in JA level was observed, its concentration remained relatively high under the effect of FR. This, in combination with the inhibition of *RhBOP1*, might contribute to a reduction in cell division.

*PLL4* belongs to a gene family involved in regulating meristem development, playing a key role in the proliferation and differentiation of stem cells and thereby ensuring the essential balance for continuous growth [56,57]. This gene family also acts outside the meristem and participates in regulating organ development. For instance, in Arabidopsis, the inhibition of *PLL1* expression led to a reduction in pedicel elongation [58].

Taken together, these findings suggest that the lack of internode elongation, i.e., the architectural stability observed in KO in response to FR, might result from a decrease in cell division, compensated for by an increase in cell elongation.

## 4. Materials and Methods

This study was carried out in Angers, France, at the experimental facilities of the Institute of Research in Horticulture and Seeds (IRHS).

### 4.1. Plant Material

The plant material consisted of two rose varieties with contrasting characteristics: ‘The Fairy’ (TF; groundcover), selected for its architectural plasticity in response to far-red (FR), and Knock Out^®^ Radrazz (KO; upright bush), chosen for its absence of response to FR [21,22].

The plants were propagated from cuttings taken from mother plants grown in pots within a greenhouse. Each cutting consisted of a single metamer collected from the middle section of the stems. The cuttings were rooted in plugs (35 mm in diameter and 40 mm in height) made from a non-woven fabric containing a mixture of fine peat and perlite. Rooting occurred in a greenhouse under a plastic tunnel, with an average temperature of 22 °C during the day and 18 °C at night, and relative humidity maintained at saturation using a fine misting system. After four weeks, the rooted cuttings were potted into 0.5 L pots containing a substrate composed of 50% peat, 40% perlite, and 10% coconut fibers and were acclimatized in the greenhouse for one week.

### 4.2. Experimental Conditions

Following the acclimation phase, the plants were grown in a climate chamber (with two shelves measuring 3.80 × 0.80 m). Mineral nutrition was supplied via fertigation, using sub-irrigation with a balanced liquid fertilizer (N-P_2_O_5_-K_2_O) 3-2-6, a pH of 6.5, and an average electrical conductivity of 1.2 mS·cm^−1^. The air temperature was maintained at 20 °C during the day and 18 °C at night, with relative humidity of 70%. The plants were exposed to a 16-h photoperiod, with an average photosynthetic photon flux density (PPFD; 400–700 nm) of 183.6 ± 12.8 µmol m^−2^ s^−1^ (equivalent to a daylight integral of 10.5 mol m^−2^ d^−1^), provided via an LED lighting system consisting of panels with 12 LED tubes emitting white, red, and far-red light (Cesbron, Saint Sylvain d’Anjou, France) according to the tested light conditions. The climate chamber was divided into two compartments, allowing the simultaneous testing of two light conditions with the following spectral characteristics: condition 1 (WR), consisting of three white LED tubes and six red LED tubes, provided 21.2 µmol·m^−2^ s^−1^ of blue (B; 11.5% of the PPFD; 400–500 nm), 30.4 µmol·m^−2^ s^−1^ of green (G; 16.5%; 500–600 nm), 132.8 µmol·m^−2^ s^−1^ of red (R; 72.0%; 600–700 nm), and 5.0 µmol·m^−2^ s^−1^ of far-red (FR; 700–800 nm), while condition 2 (WRFR), consisting of three white LED tubes, six red LED tubes, and three far-red LED tubes, provided an average of 20.4 µmol·m^−2^ s^−1^ of B (11.1%), 28.7 µmol·m^−2^ s^−1^ of G (15.7%), 133.7 µmol·m^−2^ s^−1^ of R (73.2%), and 22.0 µmol·m^−2^ s^−1^ of FR.

PPFD was measured using a LI-190 PAR meter/quantum meter (LI-COR, Lincoln, NE, USA), while the light spectrum was recorded using a Rainbow Light MR16 spectrophotometer (Rainbow Light Technology CO., Ltd., Taoyuan, Taiwan) positioned at the top of the pot. The spectral characteristics (PPFD, TPFD, YPFD, B/R, B/G, and R/FR) for each condition are detailed in Table 3. No significant differences were observed for PPFD, YPFD, B/R, and B/G between the two light conditions (Table 3).

### 4.3. Kinetics of Construction of the Order 1 Axis

Plant architecture is characterized by two components: the axis and the metamer. The metamer consists of an internode, a node, an axillary bud, and a leaf [59]. These components are topologically related to each other in succession. The kinetics of elongation of the order 1 axis, as well as the emission and elongation of its metamers, was studied by digitizing the order 1 axis using a MicroScribe 3D digitizer (Solution Technologies, Oella, MD, USA) every two to three days, starting from the 6th day of cultivation (1st, 6th, 8th, 10th, 13th, and 15th days of cultivation). The measurements were recorded in an Excel spreadsheet, allowing for the construction of an architectural database. From these data, and using a specially developed R script, the length of the order 1 axis (LA1) and the number of metamers (NbMetA1) were extracted. The average length of the metamers was then calculated by dividing the length of the order 1 axis (LA1) by its number of metamers (NbMetA1).

Measurements were taken on 10 to 12 plants per variety and light condition, sampled from the center of the shelf and monitored throughout the experiment. The experiment was carried out in triplicate.

### 4.4. Measurement of the Length of Parenchyma Cells in the Fourth Internode Underlying the Apex of the Order 1 Axis

On the 14th day of cultivation, the fourth internode underlying the apex of the order 1 axis was collected from three plants for each variety and light condition. This growing internode was chosen instead of the one underlying the apex because it was longer and, therefore, easier to handle during the subsequent treatment steps:

Fixation at 4 °C: The samples were immersed in a 4% (*v*/*v*) glutaraldehyde solution mixed with a 0.2 mol L^−1^ phosphate buffer at pH 7.2. The volume of the solution was 50 times the volume of the sample. Each sample was placed under a vacuum to remove air for 5 h and 30 min. The vacuum was interrupted every 10 min for 2 h and then every 30 min for 3 h and 30 min. The 4% (*v*/*v*) glutaraldehyde solution was then renewed. The samples were kept at 4 °C for 12 h. Finally, the samples were rinsed twice with phosphate buffer at pH 7.2 and stored at 4 °C.

Dehydration at room temperature: The samples were rinsed three times with distilled water and successively immersed in 50% (*v*/*v*) alcohol for 10 min, 70% (*v*/*v*) alcohol for 10 min, 90% (*v*/*v*) alcohol for 10 min, and 100% alcohol for 15 min.

Pre-infiltration: The samples were transferred to a pre-infiltration solution (composed of 100% alcohol and Technovit^®^ 7100 resin (Heraeus Kulzer GmbH, Wehrheim, Germany; *v*/*v*)) at 4 °C, under a vacuum for 2 h. They were then stored for 12 h at 4 °C.

Infiltration: The samples were transferred to the infiltration solution (composed of a packet of hardener I (Heraeus Kulzer GmbH, Wehrheim, Germany) dissolved in 100 mL of Technovit^®^ 7100 resin) at 4 °C, under a vacuum for at least 20 min. They were then stored for 12 h at 4 °C.

Embedding: The samples were embedded with an embedding solution composed of 1 mL of hardener II (Heraeus Kulzer GmbH, Wehrheim, Germany) and 15 mL of the infiltration solution. After 2 h of polymerization, the samples were placed in an oven at 37 °C for 12 h and then stored at 37 °C.

Longitudinal sections of 3 µm thickness were made using a Leica RM2165 microtome (Leica Microsystems Nussloch GmbH, Nussloch, Germany). The sections were then stained with 1% toluidine blue and observed with an Axio Zoom V16 macroscope (Carl Zeiss Microscopy GmbH, Oberkochen, Germany) equipped with an Axiocam 305 color camera (Carl Zeiss Microscopy GmbH, Oberkochen, Germany) at 50× magnification. The length of the parenchyma cells was measured from photographs using the Adiposoft plugin developed for Fiji, the extended version of the free and open-source image processing and analysis software, ImageJ (v. 1.50i; National Institutes of Health, Bethesda, MD, USA). The estimated vertical cell number in the internode underlying the apex was calculated by dividing the internode length by the average parenchyma cell length.

The experiment was carried out in duplicate. For each experiment, between 662 and 971 cells were measured per variety and light condition.

### 4.5. Hormone Concentration Measurements in the Internode Underlying the Apex of the Order 1 Axis

On the 14th day of cultivation, the internode underlying the apex of the order 1 axis was collected from at least 22 plants for each variety and light condition. The hormone concentration measurements (indole-3-acetic acid (IAA), cytokinins (CKs), including dihydrozeatin (DHZ), isopentenyladenine (iP), and trans-zeatin (tZ), active gibberellins, gibberellin A1 and A4 (GA1 and GA4), abscisic acid (ABA), salicylic acid (SA) and jasmonic acid (JA)) were carried out at the Plant Hormone Quantification Service, IBMCP (Valencia, Spain).

For each sample, about 6 mg of dry weight of internode was suspended in 80% methanol-1% acetic acid. Internal standards (deuterium-labeled hormones for IAA, CKs, GAs, ABA, and SA; dhJA (dihydrojasmonic acid) for JA quantification) were added and mixed by shaking for 1 h at 4 °C. The extract was kept at −20 °C overnight, then centrifuged, and the supernatant dried in a vacuum evaporator. The dry residue was dissolved in 1% acetic acid and passed through a reverse phase column (Oasis HLB; Waters Cromatografía S.A., Barcelona, Spain), as described by [60].

For IAA, GAs, ABA, SA, and JA quantification, the dried eluate was dissolved in 5% acetonitrile-1% acetic acid, and the hormones were separated using an autosampler and reverse phase UHPLC chromatography (2.6 µm Accucore RP-MS column, 100 mm length × 2.1 mm i.d.; ThermoFisher Scientific Inc., Waltham, MA, USA) with a 5 to 50% acetonitrile gradient containing 0.05% acetic acid at 400 µL min^−1^ for 21 min.

For CKs, the extracts were additionally passed through a cation-exchange column (Oasis MCX; Waters Cromatografía S.A., Barcelona, Spain) and eluted with 60% methanol-5% NH_4_OH to obtain the basic fraction containing CKs. The final eluate was dried and dissolved in 5% acetonitrile–1% acetic acid, and the CKs were separated with a 5 to 50% acetonitrile gradient for 10 min.

The hormones were analyzed with a Q-Exactive mass spectrometer (Orbitrap detector; ThermoFisher Scientific Inc., Waltham, MA, USA) via targeted Selected Ion Monitoring (SIM). The hormone concentrations in the extracts were determined using embedded calibration curves and the Xcalibur 4.0 and TraceFinder 4.1 SP1 programs.

The experiment was carried out in triplicate.

### 4.6. RNA Extraction, Illumina Sequencing, and RNA-Seq Data Analysis

On the 13th day of cultivation, the internode underlying the apex of the order 1 axis was collected from at least 22 plants for each variety and light condition. Total internode RNA was extracted using the protocol developed by [61]. The quantity and quality of the RNA were checked using a Nanodrop One (Thermo Scientific, Waltham, MA, USA) and a 2100 Bioanalyzer (Agilent Technologies, Santa Clara, CA, USA), respectively. The RNA quality was good (RNA Integrity Number, RIN = 8.5–9.7; 28S/18S ratio = 1.2–1.8). The construction of the cDNA library, sequencing on the Illumina platform, and analysis of RNAseq data (except differential expression analysis) were carried out by Novogene Company Ltd. (Cambridge, UK) as follows.

Quality control: To obtain clean reads, raw data were filtered as follows. Reads with adapter contamination, with more than 10% uncertain nucleotides (N > 10%), and with low-quality nucleotides (base quality < 5) were removed.

Mapping to the rose reference genome: Paired-end clean reads were aligned to the rose reference genome [62] using HISAT2.

Quantification of gene expression: The number of reads mapped to each gene was counted. The level of gene expression was estimated using the FPKM method (fragments per kilobase of transcript sequence per million base pairs sequenced), which takes into account the effects of sequencing depth and gene length on fragment count [63].

Analysis of genes with differential expression levels: For each variety, differential expression analysis between light conditions was performed using EdgeR in Anadiff [64]. The *p*-values obtained were adjusted using the Benjamini and Hochberg approach to control the false discovery rate (FDR). Genes with an adjusted *p*-value < 0.05 or <0.1 and a log_2_ fold change (log_2_ FC) ≥ 1 or ≤−1 were considered differentially expressed.

The experiment was carried out in triplicate.

### 4.7. Real-Time Quantitative Polymerase Chain Reaction (qRTPCR)

Differential expression analysis was confirmed via quantitative real-time reverse transcription-PCR (qRT-PCR). Four differentially expressed genes (*RhMYB73*, *RhRBK2*, *RhBOP1,* and *RhGA2ox*) were selected. cDNAs were obtained through reverse transcription performed on 1 μg of RNA using SuperScript III Reverse Transcriptase (Invitrogen, Waltham, MA, USA). qRT-PCR was performed with SYBR Green Supermix (Bio-Rad, Hercules, CA, USA) using cDNA as a template, following the protocol from [65]. Relative gene expression was calculated using the 2^−ΔΔCt^ method, with *RhUBC* as the internal control [66,67]. Each PCR reaction was carried out in triplicate (three biological and technical replicates).

### 4.8. Data Analysis

For each variety, the effect of FR was evaluated for all variables. A mixed linear model was used, with the replication of the experiment as a random factor, for a probability of *p* < 0.05.

The model was estimated using the maximum likelihood (ML) method. A post hoc comparison of means (Tukey’s test) was performed, using the adjusted means (least-square means), for a probability of *p* < 0.05. Statistical analyses were carried out using the lme4 and multcomp packages in R (R Foundation for Statistical Computing, Vienna, Austria).

## 5. Conclusions

**Figure 5:**
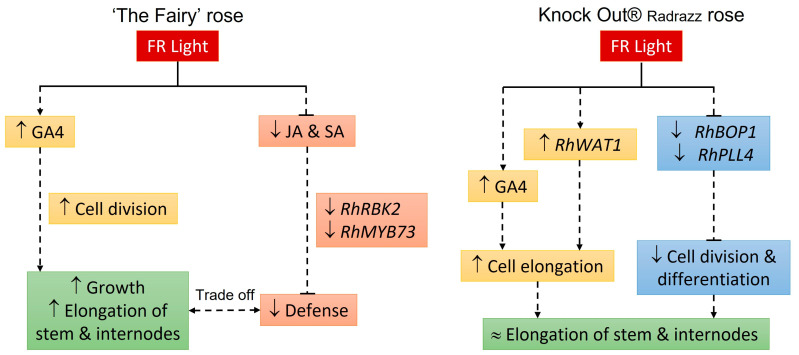
Schematic diagram illustrating the mechanisms of internode elongation in response to far-red (FR) in ‘The Fairy’ (TF) and Knock Out^®^ Radrazz (KO). In TF, FR induced an increase in the concentration of gibberellin A4 (GA4), which stimulated cell division, while the concentrations of jasmonic acid (JA) and salicylic acid (SA) decreased, thereby reducing the plant’s defensive capabilities in favor of growth, specifically internode elongation. In KO, FR promoted the increase in GA4 concentration, as well as the expression of the *RhWAT1* gene, stimulating cell elongation. Meanwhile, the inhibition of *RhBOP1* and *RhPLL4* gene expression limited cell division and differentiation. This inhibition, combined with the increased cell elongation, prevented internode elongation. Regular arrow, positive effect; inverted T, negative effect.

This study highlighted significant differences in the regulatory mechanisms of internode elongation in rose in response to far-red (FR; Figure 5). For ‘The Fairy’ (TF), the elongation of internodes was primarily attributed to an increase in cell division, facilitated via elevated levels of gibberellins (GAs), particularly gibberellin A4 (GA4), and a decrease in jasmonic acid (JA) concentration. Additionally, the reduction in the plant’s defensive capabilities, resulting from decreased levels of JA and salicylic acid (SA), might promote growth by reallocating carbon resources toward elongation rather than defense mechanisms. This trade-off between growth and defense illustrates TF’s adaptability to its environment, as has been repeatedly observed [6,8,21,22]. In contrast, in Knock Out^®^ Radrazz (KO), although the cell length increased, internode elongation was not observed due to a decrease in the cell number, which might be behind the architectural stability. This response was correlated with a high level of GA4, as well as the inhibition of *Block of Cell Proliferation 1* (*BOP1*), a positive regulator of cell division. Thus, this study highlighted the complexity of the physiological responses in rose. Expanding it to include additional varieties could further enhance the understanding of the diversity of FR responses. By tapping into this diversity of responses, particularly phenotypic stability, it might be possible to develop an innovative approach to obtain plants with the desired architecture, regardless of environmental conditions.

## Figures and Tables

**Table 1:** The least-square means (LS means) of parenchymal cell length in the fourth internode underlying the apex of the order 1 axis on the 14th day of cultivation and estimated vertical cell number in the internode underlying the apex of the order 1 axis on the 13th day of cultivation for Knock Out^®^ Radrazz (KO) and ‘The Fairy’ (TF) grown in a climate chamber under two light conditions: WR and WRFR.

Genotype	Fourth Internode Underlying the Apex on the 14th Day of Cultivation		Internode Underlying the Apex on the 13th Day of Cultivation	
	Cell Length (µm)		Estimated Vertical Cell Number	
	WR	WRFR	WR	WRFR
KO	21.4 a	23.7 b	283.0	236.0
TF	20.9 a	21.4 a	173.0	233.0

Means followed by the same lowercase letter in the same line are not significantly different (Tukey’s test, *p* < 0.05).

**Table 2:** Genes differentially expressed in the internode underlying the apex of the order 1 axis for Knock Out^®^ Radrazz (KO) and ‘The Fairy’ (TF) grown in a climate chamber for 13 days under two light conditions: WR and WRFR.

DEGs	Gene ID	Description	Name	KO_WRFR vs. KO_WR		
				log_2_ Fold Change	*p*-Value	Adjusted *p*-Value (FDR) ^1^
Down-regulated	RC7G0576300	Block of cell proliferation 1	*BOP1*	−1.0^2^	4.4 × 10^−7^	**7.0 × 10^−3^**
	RC5G0246800	Gibberellin 2-beta-dioxygenase 1	*GA2ox1*	−1.0	2.5 × 10^−6^	**2.0 × 10^−2^**
	RC5G0037300	Gibberellin 2-beta-dioxygenase 8	*GA2ox8*	−1.6	1.9 × 10^−5^	9.0 × 10^−2^
	RC3G0259000	Poltergeist-like	*POL-like*; *PLL4*	−1.8	2.5 × 10^−5^	9.0 × 10^−2^
	RC6G0422400	S-adenosyl-L-methionine-dependent tRNA 4-demethylwyosine synthase	*TYW1*	−2.5	2.1 × 10^−5^	9.0 × 10^−2^
Up-regulated	novel,2696	Prolyl-tRNA synthetase	*PRORS1*	2.5	7.5 × 10^−7^	**8.0 × 10^−3^**
	RC0G0173400	Walls are thin 1 related protein	*WAT1*	2.5	2.6 × 10^−5^	9.0 × 10^−2^
**DEGs**	**Gene ID**	**Description**	**Name**	**TF_WRFR vs. TF_WR**		
				**log_2_ Fold Change**	***p*-Value**	**Adjusted *p*-Value (FDR)**
Down-regulated	RC6G0094600	Transcription factor	*MYB73*	−1.0	6.4 × 10^−6^	**3.0 × 10^−2^**
	RC4G0261800	Receptor-like cytosolic serine/ threonine-protein kinase	*RBK2*	−1.2	9.4 × 10^−6^	**4.0 × 10^−2^**
	RC6G0474700	Uncharacterized protein	*Y1015*	−3.6	5.8 × 10^−8^	**9.0 × 10^−4^**
Up-regulated	RC6G0451600	Nucleoid-associated protein	*STIC2*	2.8	4.6 × 10^−6^	**3.0 × 10^−2^**

^1^ FDR stands for false discovery rate. Bold values indicate the significantly differentially expressed genes (DEG) at an adjusted *p*-value (FDR) < 0.05.

**Table 3:** Spectral characteristics of the two light conditions, WR and WRFR.

Light Conditions	PPFD (µmol m^−2^ s^−1^)	TPFD (µmol m^−2^ s^−1^)	YPFD (µmol m^−2^ s^−1^)	B/R	B/G	R/FR
WR	184.4 a ^1^	189.4 a	165.8 a	0.2 a	0.7 a	26.6 b
WRFR	182.8 a	204.8 b	166.5 a	0.2 a	0.7 a	6.1 a

PPFD, photosynthetic photon flux density (400–700 nm); TPFD, total photon flux density (400–800 nm); YPFD, yield photon flux density, i.e., the product of TPFD and relative quantum efficiency; B/R, blue light (400–500 nm) over red light (600–700 nm) ratio; B/G, blue light (400–500 nm) over green light (500–600 nm) ratio; R/FR, red light (600–700 nm) over far-red light (700–800 nm) ratio. ^1^ Means followed by the same lowercase letter in the same column are not significantly different (Kruskall & Wallis test, *p* < 0.05).

## Data Availability

The original contributions presented in this study are included in the article. Further inquiries can be directed to the corresponding author.
